# The corticosteroid compounds prednisolone and vamorolone do not alter the nociception phenotype and exacerbate liver injury in sickle cell mice

**DOI:** 10.1038/s41598-018-24274-6

**Published:** 2018-04-17

**Authors:** Luis E. F. Almeida, Jesse M. Damsker, Sarah Albani, Nina Afsar, Sayuri Kamimura, Drew Pratt, David E. Kleiner, Martha Quezado, Heather Gordish-Dressman, Zenaide M. N. Quezado

**Affiliations:** 1 Department of Perioperatice Medicine, National Institutes of Health Clinical Center, National Institutes of Health, Besthesda, MD USA; 2 Sheikh Zayed Institute for Pediatric Surgical Innovation, Children’s Research Institute, Children’s National Health System, Washington, DC USA; 3 Reveragen BioPharma Inc., Rockville, MD USA; 4 Laboratory of Pathology, National Cancer Institute, National Institutes of Health, Besthesda, MD USA; 5 Department of Pathology, University of Michigan, Ann Arbor, MI USA; 6 Center for Genetic Medicine Research, Children’s Research Institute, Children’s National Health System, Department of Integrative Systems Biology, George Washington University School of Medicine and Health Sciences, Washington, DC USA

## Abstract

Clinicians often hesitate prescribing corticosteroids to treat corticosteroid-responsive conditions in sickle cell disease (SCD) patients because their use can be associated with complications (increased hospital readmission, rebound pain, strokes, avascular necrosis, acute chest syndrome). Consequently, SCD patients may receive suboptimal treatment for corticosteroid-responsive conditions. We conducted a preclinical trial of dissociative (vamorolone) and conventional (prednisolone) corticosteroid compounds to evaluate their effects on nociception phenotype, inflammation, and organ dysfunction in SCD mice. Prednisolone and vamorolone had no significant effects on nociception phenotype or anemia in homozygous mice. Conversely, prednisolone and vamorolone significantly decreased white blood cell counts and hepatic inflammation. Interestingly, the effects of vamorolone were milder than those of prednisolone, as vamorolone yielded less attenuation of hepatic inflammation compared to prednisolone. Compared to controls and heterozygotes, homozygotes had significant liver necrosis, which was significantly exacerbated by prednisolone and vamorolone despite decreased hepatic inflammation. These hepatic histopathologic changes were associated with increases in transaminases and alkaline phosphatase. Together, these results suggest that, even in the setting of decreasing hepatic inflammation, prednisolone and vamorolone were associated with significant hepatic toxicity in SCD mice. These findings raise the possibility that hepatic function deterioration could occur with the use of corticosteroids (conventional and dissociative) in SCD.

## Introduction

In sickle cell disease (SCD), a single point mutation in the β globin gene generates a variant form of hemoglobin (hemoglobin S), which, as a result of alteration in its tertiary structure, can polymerize during deoxygenation. In turn, polymerization of hemoglobin S damages erythrocyte membranes, leads to hemolysis and triggers a cascade of pathologic events including endothelial dysfunction, inflammation, increased adhesion of neutrophils and platelet activation, vaso-occlusion, ischemia/reperfusion injury, hypercoagulability, and oxidative stress^1^. These events have been shown to underlie clinical complications of SCD including acute and chronic pain, strokes, acute chest syndrome, as well as liver, kidney, cardiac, and pulmonary dysfunction^1^. Unfortunately, despite an increased understanding of the pathobiology of SCD, there is a paucity of disease-modifying therapies and as a result, patients endure significant life-long morbidity and decreased life-expectancy^1,2^. Therefore, the development of therapies targeting the mechanisms underlying these SCD complications are needed.

Ample evidence indicates that inflammation greatly contributes to the complications of SCD^3^. During steady state, SCD patients often have ongoing inflammation indicated by elevated white cell counts and increased levels of inflammatory mediators (interleukins, tumor necrosis factor, and adhesion molecules)^4–6^. During vaso-occlusive episodes (VOE), the most common complication and the reason for most hospital admissions in SCD patients, this ongoing inflammatory state worsens, which contributes to vascular occlusion, ischemia/reperfusion injury, and acute pain^5,7^. Acute chest syndrome, another feared complication and a significant cause of morbidity and mortality in SCD^8,9^, is also associated with upregulation of cytokines, increased white blood cells adherence, and resulting vascular and pulmonary damage^10,11^. Given the role of inflammation in SCD-associated morbidities, researchers have evaluated the use of corticosteroids to treat VOEs and acute chest syndrome^12,13^. Small clinical trials in children have shown that a short course of methylprednisolone decreases the duration of severe pain and opioid requirements^14,15^. In patients admitted with acute chest syndrome, a course of dexamethasone decreased hospitalization time^16,17^. However, corticosteroid use in SCD is associated with complications, which can occur both during its administration and after its discontinuation including avascular necrosis, rebound pain, stroke, and acute chest syndrome^12,18,19^. Fearing these complications, clinicians often hesitate to administer corticosteroids to SCD patients^20^. However, this reluctance in using corticosteroids limits therapeutic options and may result in suboptimal treatment of corticosteroid-responsive conditions such as asthma, acute chest syndrome, and auto-immune pathologies, which are common in SCD. Therefore, development of new corticosteroid compounds that retain the desirable anti-inflammatory properties and have less of the undesirable side-effects, could provide a potential solution to mitigate the reported serious corticosteroid-related side effects in SCD.

The anti-inflammatory effects of corticosteroids are believed to be due to transrepression and their side-effects to transactivation of gene transcription. Vamorolone (ReveraGen BioPharma, Rockville, MD) is a new dissociative corticosteroids compound, which has been optimized to preferentially induce transrepression, thus retaining anti-inflammatory properties and to minimize transactivation, thus minimizing undesirable side-effects and resulting in a milder side effect profile compared to conventional corticosteroids^21–24^. In mouse models of muscle dystrophy, experimental autoimmune encephalomyelitis, colitis, and cortical brain tumors, vamorolone has been shown to increase cellular membrane stability, to improve disease-related symptoms and to have fewer side-effects (e.g. stunted growth, hormonal imbalance, immunosuppression) compared with conventional corticosteroids^22–26^. Furthermore, vamorolone was shown to be well tolerated in healthy adults and is currently in phase 2 clinical trials in patients with Duchenne muscular dystrophy.

We conducted a preclinical trial of vamorolone, a dissociative, and of prednisolone, a conventional corticosteroid, to test the hypotheses that in humanized SCD mice^27–30^ dissociative corticosteroids would improve the nociception phenotype, decrease signs of inflammation, and improve organ dysfunction. We designed a preclinical trial of vamorolone and prednisolone enrolling humanized Townes sickle cell male and female mice of all genotypes [controls, heterozygotes, and homozygotes (sickling)]. Homozygotes in this mouse strain are known to recapitulate hematologic and nociception phenotypes of human SCD^27–32^.

## Results

The number of animals enrolled in each experimental group is shown on the Table 1. All mice completed the treatment and outcome measurements except for four homozygotes and one heterozygote treated with vamorolone, three homozygotes, one heterozygote, and one control mouse treated with prednisolone, and one homozygote and one control mouse treated with vehicle, which died before trial completion.

**Table 1:** Number of animals entering the study for each experimental group*.

Genotype	Treatment		
	Vehicle	Prednisolone (30 mg/kg)	Vamorolone (30 mg/kg)
Controls	16	16	15
Heterozygotes	16	18	22
Homozygotes	15	17	19

*Male and female mice were treated orally with vehicle, prednisolone, or vamorolone daily for six weeks.

### Effect of prednisolone and vamorolone on nociception

**Figure 1:**
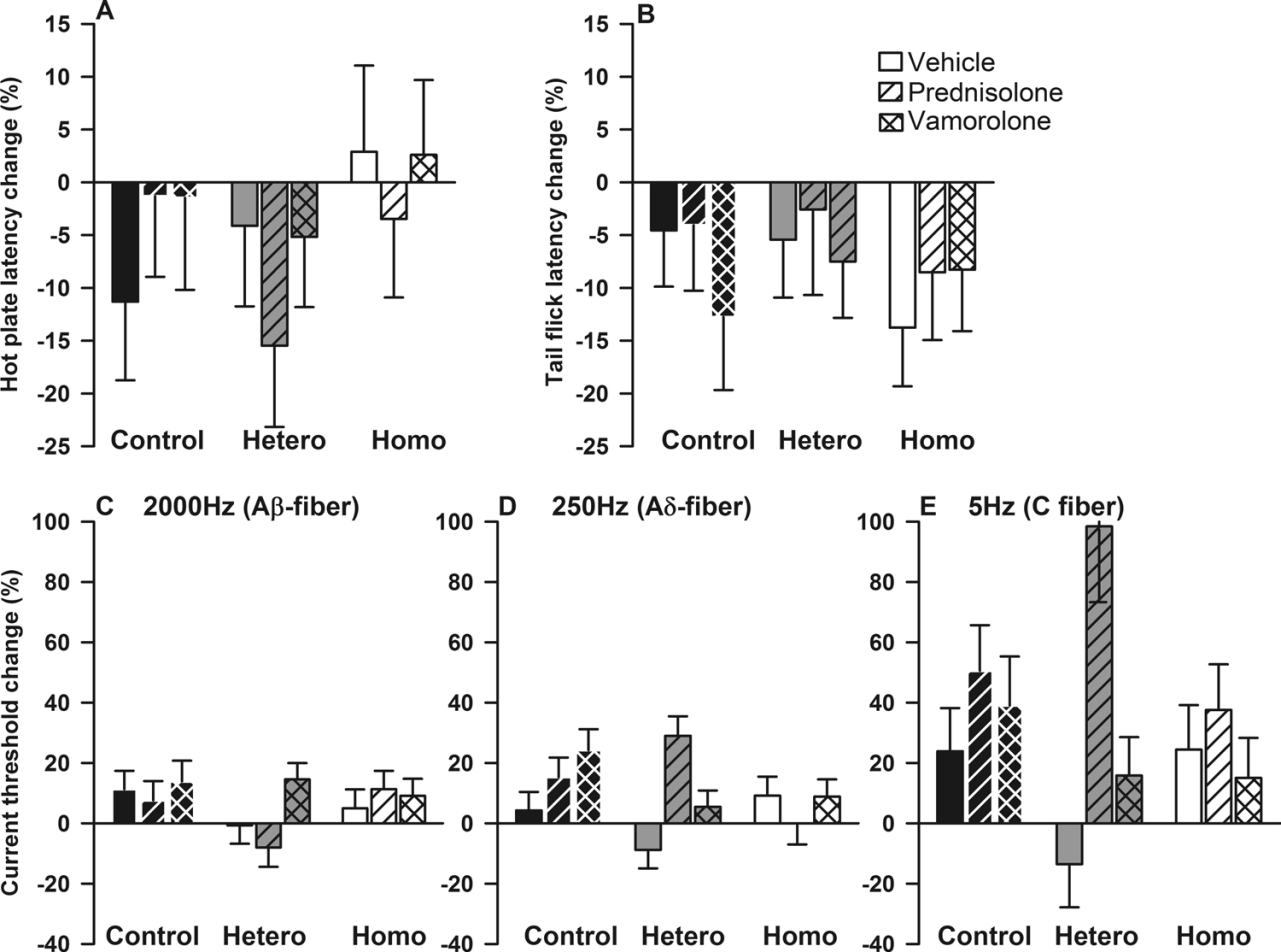
Effect of prednisolone and vamorolone on nociception. Data are presented as means and standard errors of percentage changes from baseline by genotype and treatment for hot plate (**A**) and tail flick latencies (**B**), current thresholds in response to 2000 (**C**), 250 (**D**) or 5 Hz (**E**) stimulations. Hetero indicates heterozygotes and homo homozygotes (N = 13–18 per each of 9 treatment groups for all outcome measurements). Regardless of genotype and sex, six-week treatment with vehicle, prednisolone, or vamorolone yielded similar percent changes from baseline on hot plate and tail flick latencies and on current thresholds in response to 2000 or 5 Hz stimulations (all p > 0.05, **A**,**B**,**C** and **E**). Regarding 250 Hz current thresholds, the effects of prednisolone and vamorolone, compared with vehicle, varied according to genotype as there was a significant treatment*genotype interaction (p = 0.014) although neither the main effect of treatment or genotype reached statistical significance (p = 0.07 and p = 0.18, respectively, (**D**). As shown in (**D**), the pattern of percent change from baseline over treatment groups is markedly different for each genotype.

In order to evaluate the effects of the six-week treatment (prednisolone, vamorolone, or vehicle) according to genotype, we examined thermosensory response and current vocalization thresholds percent changes from baseline. Overall, regardless of genotype and sex, six-week treatment with vehicle, prednisolone, or vamorolone yielded similar percent changes from baseline on hot plate and tail flick latencies and on current thresholds in response to 2000 or 5 Hz stimulations (all p > 0.05, Fig. 1A,B,C and E). Regarding 250 Hz current thresholds, the effects of prednisolone and vamorolone, compared with vehicle, on changes from baseline varied according to genotype as there was a significant treatment*genotype interaction (p = 0.014) although neither the main effect of treatment or genotype reached statistical significance (p = 0.07 and p = 0.18, respectively). As shown in Fig. 1D, the pattern of percent change from baseline over treatment groups is markedly different for each genotype. Whereas control mice showed increasing percent change from baseline with vehicle, prednisolone, and vamorolone treatments, heterozygous and homozygous mice showed a significantly different pattern of effect in response to the experimental treatments.

### Effect of prednisolone and vamorolone on hematologic parameters and spleen size

**Figure 2:**
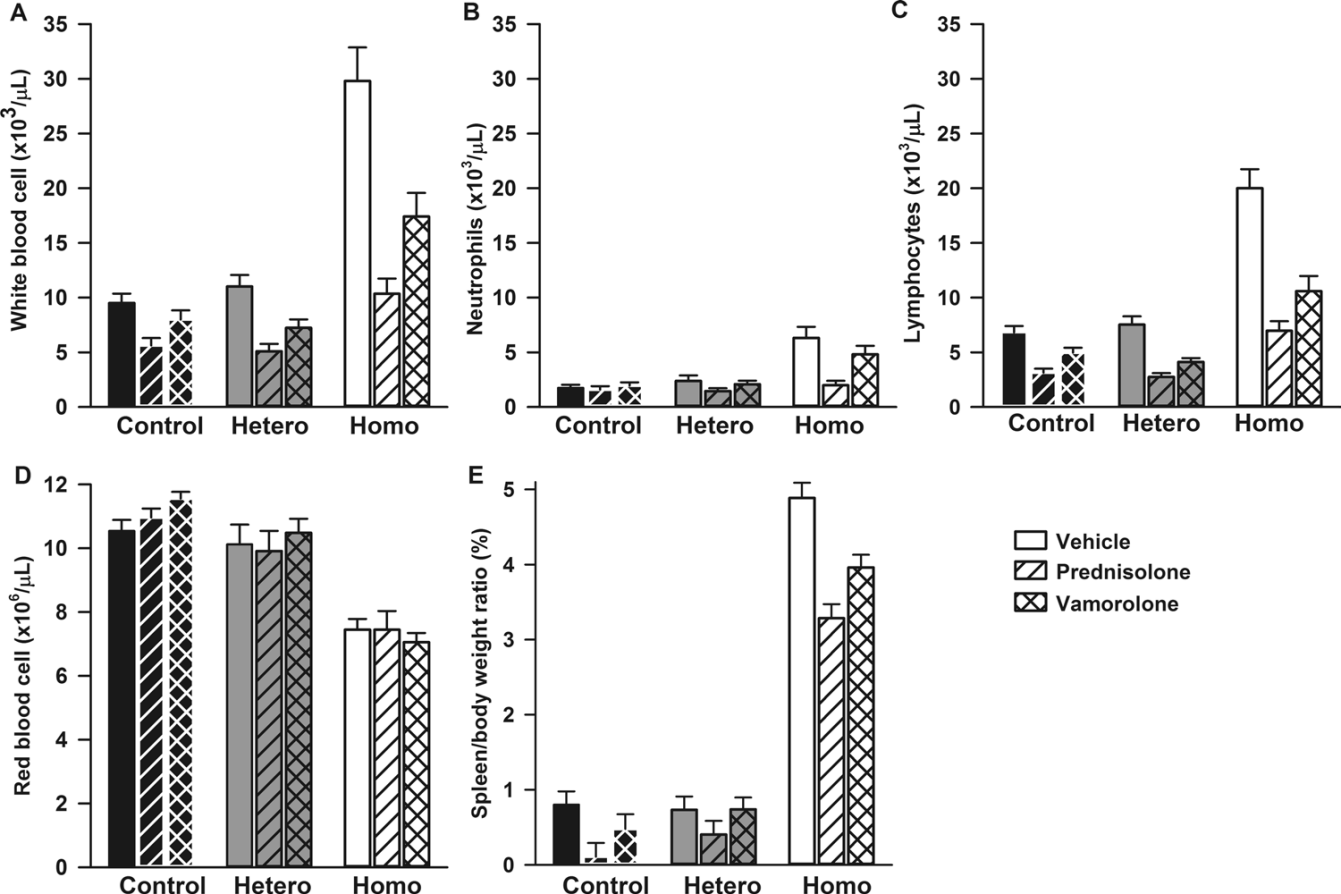
Effect of prednisolone and vamorolone on hematologic parameters and spleen size. Data are presented as means and standard errors. Hetero indicates heterozygotes and homo homozygotes (N = 13–17 per each of 9 treatment groups for all outcome measurements). (**A**) Homozygous mice, independent of treatment, had increased white blood cell counts p < 0.001 compared to control animals. Regardless of genotype, prednisolone- and vamorolone-treated mice had significantly lower white blood cell counts compared with vehicle-treated animals (p < 0.001). The effects of treatment on the number of neutrophils varied according to genotype (p = 0.045 for the treatment*genotype interaction term). (**B**) The number of neutrophils was significantly higher in homozygous mice as compared to controls (p = 0.006) and heterozygous mice (p = 0.006) for all treatment groups, however no main effect of treatment was observed (p = 0.21). (**C**) Homozygous mice had significantly greater lymphocytes than either control (p < 0.001) or heterozygous mice (p < 0.001) and both, prednisolone (p = 0.027) and vamorolone-treatment (p < 0.001) were associated with decreased lymphocyte counts compared with vehicle. (**D**) Homozygotes, independent of treatment, had lower red cell counts, compared to controls and heterozygotes (p < 0.001). Treatment with prednisolone and vamorolone had no effect on red cell counts, regardless of genotype (p = 0.69). (**E**) Homozygous mice had significantly increased spleen-to-body weight ratio compared with heterozygotes and control animals (all p < 0.001) independent of treatment although the effects of treatment varied according to genotype (p = 0.010 for treatment*genotype interaction). Mice of all genotypes treated with prednisolone had significantly lower spleen to body weight ratios than vehicle treated mice (p = 0.006), however no significant difference was observed between vamorolone and vehicle treated mice independent of genotype.

Figure 2 shows hematologic parameters measured after six-week treatment with vehicle, prednisolone, or vamorolone according to genotype. Similar to previous reports^27–30^, homozygous mice, independent of treatment, had increased white blood cell counts (p < 0.001, Fig. 2A) and anemia as indicated by lower red blood cell counts (Fig. 2D), hemoglobin, and hematocrit (data not shown, all p < 0.001) compared to control animals (Fig. 2). Regardless of genotype, prednisolone- and vamorolone-treated mice had significantly lower white blood cell counts compared with vehicle-treated animals (p < 0.001, Fig. 2A). The effects of treatment on the number of neutrophils varied according to genotype (p = 0.045 for the treatment*genotype interaction term). The number of neutrophils was significantly higher in homozygous mice as compared to controls (p = 0.006) and heterozygous mice (p = 0.006) for all treatment groups (Fig. 2B), however no main effect of treatment was observed (p = 0.21). Both prednisolone (p = 0.027) and vamorolone-treatment (p < 0.001) were associated with decreased lymphocyte counts compared with vehicle. In addition, homozygous mice had significantly greater lymphocyte counts than either control (p < 0.001) or heterozygous mice (p < 0.001) independent of treatment.

There were no significant differences in red cell counts, hemoglobin, hematocrit level, or platelet counts with respect to treatment (p = 0.69, p = 0.11, p = 0.17, p = 0.84 respectively; Fig. 2D). Similarly, there was no effect of prednisolone or vamorolone on mean corpuscular volume, mean corpuscular hemoglobin, mean corpuscular hemoglobin concentration, or red cell distribution width, all p ≥ 0.08 (data not shown).

In SCD mice, the spleen is a site of extra medullary hematopoiesis. In keeping with our previous reports^30^, homozygous mice had significantly increased spleen-to-body weight ratio compared with heterozygotes and control animals (all p < 0.001) independent of treatment although the effects of treatment varied according to genotype (p = 0.010 for treatment*genotype interaction). Mice of all genotypes treated with prednisolone had significantly lower spleen to body weight ratios than vehicle treated mice (p = 0.006), however no significant difference was observed between vamorolone and vehicle treated mice (Fig. 2E) independent of genotype.

### Effect of prednisolone and vamorolone on liver function tests and malondialdehyde

**Figure 3:**
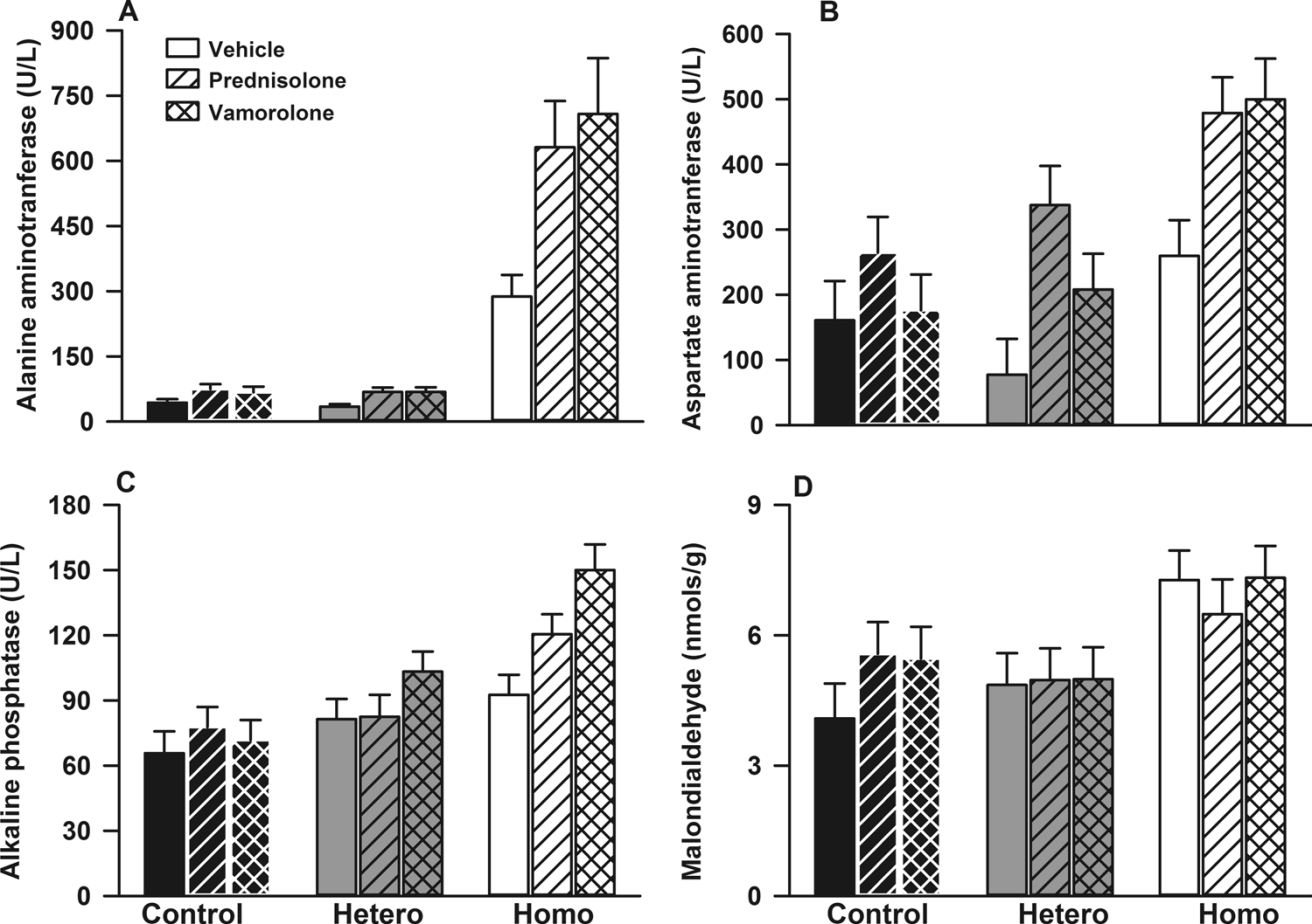
Effect of prednisolone and vamorolone on liver function tests and malondialdehyde levels. Data are presented as means and standard errors of alanine aminotransferase (ALT, **A**), aspartate aminotransferase (AST, **B**), alkaline phosphatase (ALK, **C**), and malondialdehyde (**D**). Hetero indicates heterozygotes and homo homozygotes (N = 5–7 per each of 9 treatment groups for all outcome measures). Among vehicle-treated mice, compared with controls, homozygotes had significantly higher plasma levels of ALT (p < 0.001, **A**), AST (p = 0.001, **B**), and ALK (p < 0.001, **C**). Overall, the effects of vamorolone and prednisolone on ALK, ALT, and AST were similar (p = 0.071, p = 0.27, p = 0.40 respectively). Additionally, independent of genotype, compared with vehicle, vamorolone and prednisolone treatment was associated with increased ALK and AST (p = 0.018 and p = 0.042 respectively for main treatment effect). (**D**) Homozygotes had higher levels of liver malondialdehyde formation compared with control and heterozygous mice (p < 0.001). Additionally, independent of genotype, there was no effect of treatment on malondialdehyde formation in liver homogenates (p = 0.66).

Among vehicle-treated mice, compared with controls, homozygotes had significantly higher plasma levels of alanine aminotransferase (ALT, p < 0.001), aspartate aminotransferase (AST, p = 0.001), and alkaline phosphatase (ALK, p < 0.001), Fig. 3. Overall, the effects of vamorolone and prednisolone on ALK, ALT, and AST were similar (p = 0.071, p = 0.27, p = 0.40 respectively). Additionally, independent of genotype, compared with vehicle, vamorolone and prednisolone treatment was associated with increased ALK and AST (p = 0.018 and p = 0.042 respectively for main treatment effect).

In order to examine levels of oxidative stress and lipid peroxidation, we measured malondialdehyde formation in liver homogenates. Homozygotes had higher levels of liver malondialdehyde formation compared with control and heterozygous mice (p < 0.001, Fig. 3D). However, in liver homogenates, there was no effect of treatment (prednisolone or vamorolone) compared with vehicle on malondialdehyde formation independent of genotype (p = 0.66)

### Effect of prednisolone and vamorolone on liver histopathology

**Figure 4:**
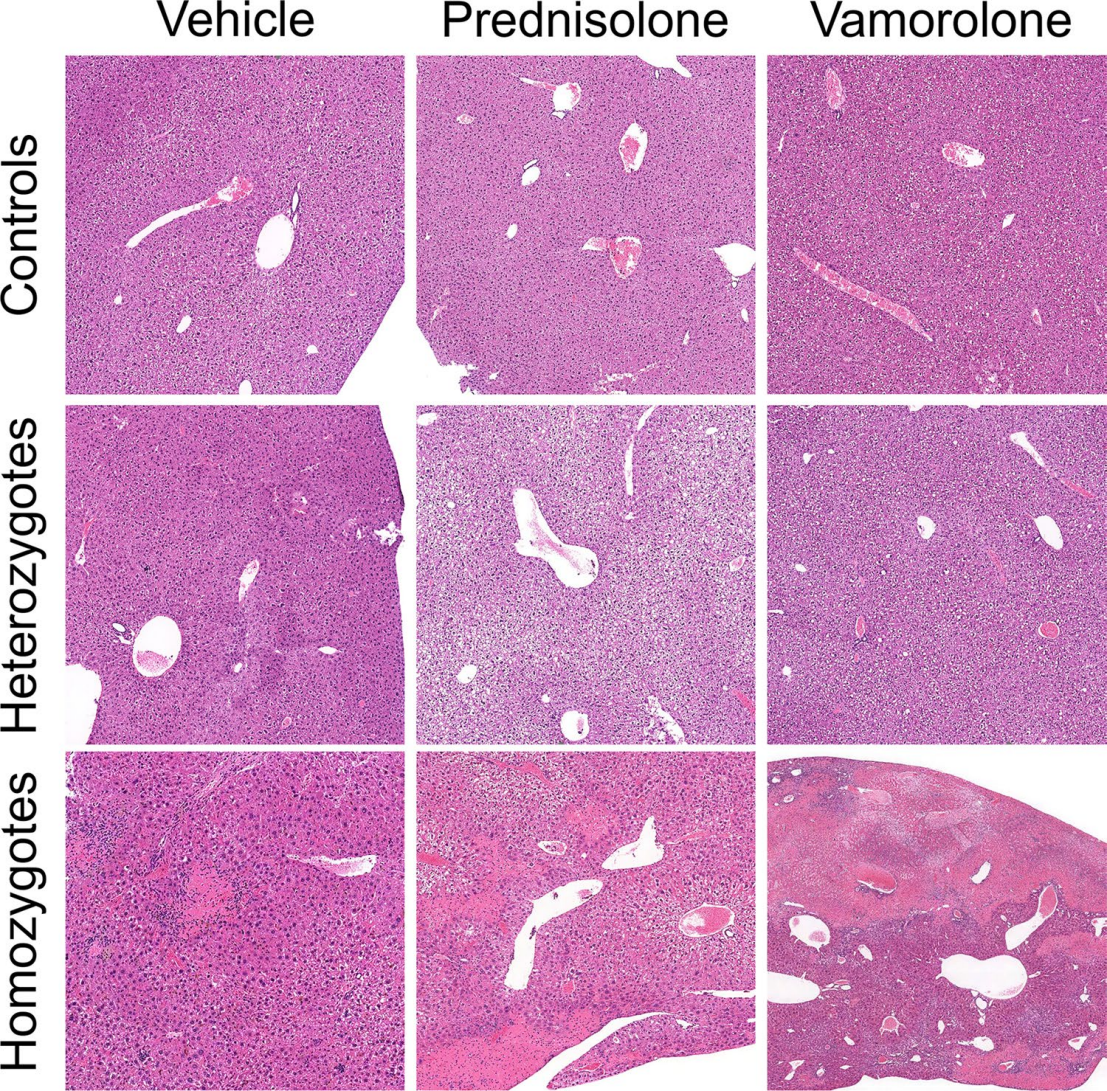
Effect of prednisolone and vamorolone on liver histopathology. Representative hematoxylin and eosin-stained sections from each genotype and respective treatment group (N = 14–20 per each of 9 treatment groups). In contrast to controls and heterozygotes, homozygous mice had significant hepatic necrosis (top row: Vehicle/Prednisolone/Vamorolone: 5x; middle row: Vehicle/Prednisolone/Vamorolone: 5x; bottom row; Vehicle: 10x, Prednisolone: 4x, Vamorolone: 3x). In homozygotes, hepatic necrosis was characterized by areas of patchy, often confluent, well-demarcated zone III (centrilobular) coagulative necrosis with admixed hemosiderin deposition and inflammation. Pools of sickled red blood cells were readily identified within the hepatic vasculature, leading to congestion and occasional occlusion within areas of necrosis.

**Figure 5:**
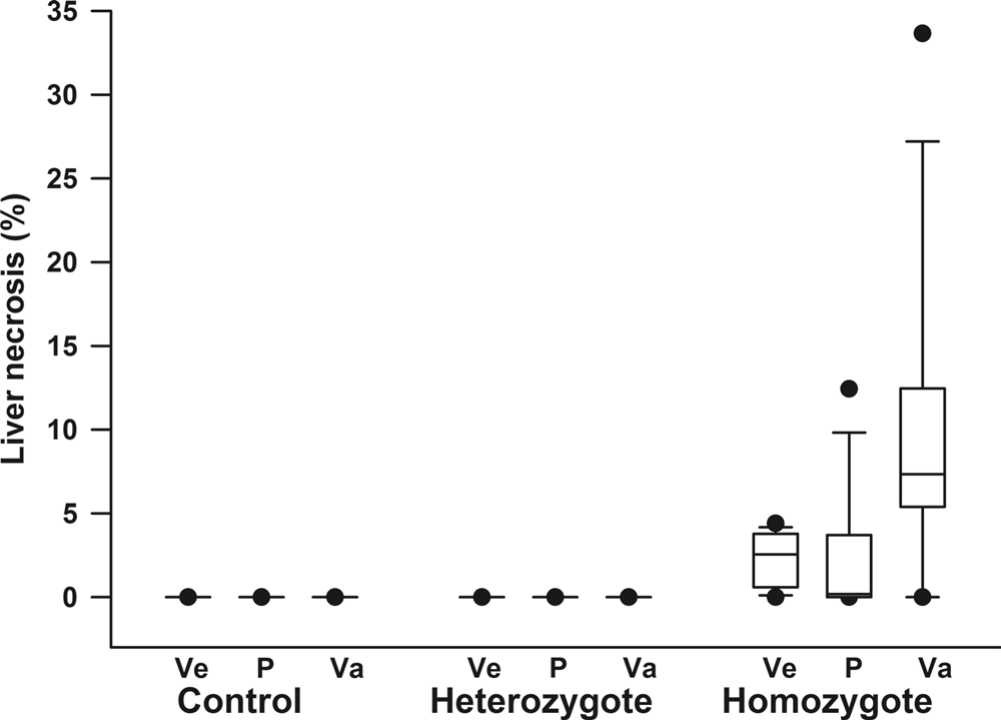
Effect of prednisolone and vamorolone on quantitative hepatic necrosis. Box plots show median, interquartile range, whiskers show 10^th^ and 90^th^ percentiles and points reflect 5^th^ and 95^th^ percentiles of quantitative liver necrosis in all experimental groups according to genotype and treatment. Ve represents vehicle, P prednisolone, and Va vamorolone. (N = 14–20 per each of 9 treatment groups). When comparing the percentage of hepatic necrosis between treatments in homozygous mice, a significant increase in hepatic necrosis was observed in vamorolone treated homozygotes compared to both vehicle- (p = 0.005) and prednisolone-treated (p = 0.008) animals. There was also a trend towards increased liver necrosis in prednisolone-treated homozygotes compared to vehicle-treated mice even though this increase in hepatic necrosis did not reach statistical significance (p = 0.15).

Hepatic necrosis was observed in homozygotes but not in controls or heterozygotes. Hepatic necrosis was characterized by areas of patchy, often confluent, well-demarcated zone III (centrilobular) coagulative necrosis with admixed hemosiderin deposition and inflammation, Fig. 4. Pools of sickled red blood cells were readily identified within the hepatic vasculature, leading to congestion and occasional vascular occlusion within areas of necrosis. Some homozygous mice showed mild to moderate hepatic glycogenosis. When comparing the percentage of hepatic necrosis between treatments in homozygous mice, a significant increase in hepatic necrosis was observed in vamorolone treated homozygotes compared to both vehicle- (p = 0.005) and prednisolone-treated (p = 0.008) animals, Fig. 5. There was also a trend towards increased liver necrosis in prednisolone-treated homozygotes compared to vehicle-treated mice even though this increase in hepatic necrosis did not reach statistical significance (p = 0.15), Fig. 5.

**Figure 6:**
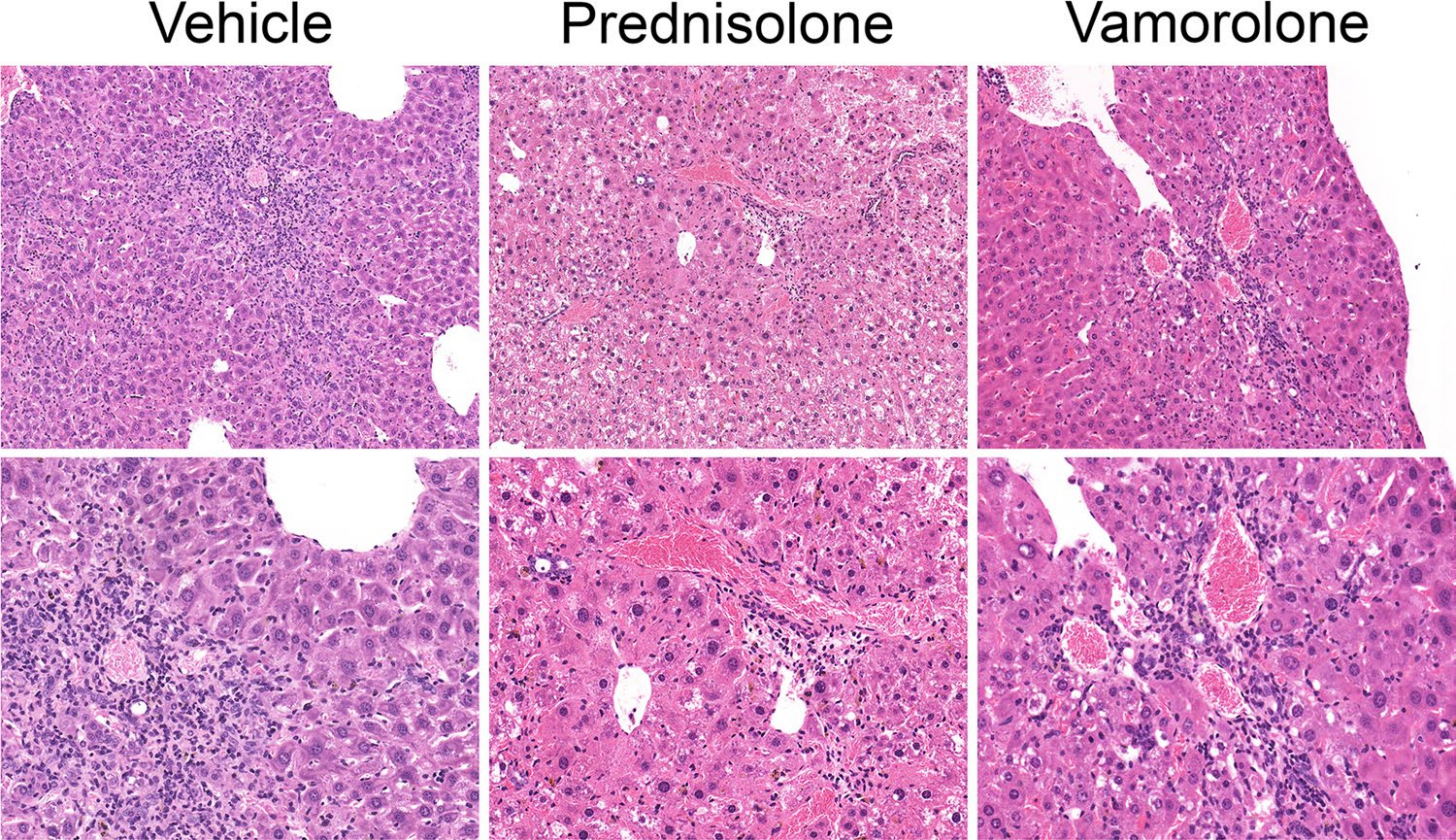
Effect of prednisolone and vamorolone on hepatic inflammation in homozygous sickle cell mice. Representative hematoxylin and eosin (H&E) slides from homozygotes shown in original magnification 10x (top row) and 20x (bottom row). N = 14–20 per each of 9 treatment groups. There was evidence of significant hepatic inflammation in homozygotes and the degree of inflammation varied between treatment groups. The infiltrates were composed predominantly of lymphocytes and macrophages, with only rare plasma cells and eosinophils. Neutrophils were found associated mainly with areas of necrosis. The degree of inflammation was worst in vehicle-treated homozygotes, less intense in vamorolone-treated and with the mildest degree seen in prednisolone-treated homozygotes.

There was also evidence of significant hepatic inflammation in homozygotes and the degree of inflammation varied between treatment groups. The infiltrates were composed predominantly of lymphocytes and macrophages, with only rare plasma cells and eosinophils. Neutrophils were found associated mainly with areas of necrosis. The degree of inflammation was worst in vehicle-treated homozygotes, less intense in vamorolone-treated and with the mildest degree seen in prednisolone-treated homozygotes, Fig. 6.

## Discussion

We conducted a preclinical trial of prednisolone, a conventional corticosteroid, and of vamorolone, a novel dissociative corticosteroid, to determine their effects on the nociception phenotype, hematologic changes, and organ pathology in humanized SCD mice. Prednisolone and vamorolone had no significant effect on the nociception phenotype, red cell count, or hemoglobin levels in SCD mice compared to vehicle. Conversely, both prednisolone and vamorolone significantly decreased white blood cell counts and the effects of vamorolone were milder than those of prednisolone. A similar pattern of effect was observed in liver histopathology as, while both vamorolone and prednisolone decreased hepatic inflammation, vamorolone attenuated hepatic inflammation to a lesser degree than did prednisolone. However, despite decreasing hepatic inflammation, prednisolone and vamorolone treatments were associated with increased hepatic necrosis in homozygotes, but not in controls and heterozygotes. However, while worsened hepatic necrosis was only observed in homozygotes, prednisolone and vamorolone were associated with significant increases in plasma levels of transaminases and alkaline phosphatase and no changes in liver levels of malondialdehyde formation in all genotypes. These findings suggest that the corticosteroids prednisolone and vamorolone, despite decreasing hepatic inflammation and not changing levels of lipid oxidative stress, were associated with significant hepatic toxicity in SCD mice.

The administration of corticosteroid compounds to SCD patients can be associated with a number of complications^18,33^. During corticosteroids administration, adverse events such as severe VOEs, hemorrhagic stroke, and death have been reported in SCD patients^18,33^. Clinicians have also reported that after completion of a course of corticosteroids and discontinuation of those drugs, SCD patients are at increased risk for hospital readmissions for VOEs^12,14,15,19^. While the mechanisms of these reported complications are largely unknown, results from preclinical studies have led to some hypotheses^34^. For example, in a different mouse model of SCD, a short course of the corticosteroid dexamethasone was shown to inhibit the nuclear factor kappa B (NF-ĸB), a pro-inflammatory transcription factor, and to decrease the expression of the vascular cell adhesion molecule-1 (VCAM-1) and the intercellular adhesion molecule-1 (ICAM-1) in lungs, liver, and skin^34^. Those effects in turn were shown to prevent hypoxia/reoxygenation-related decreases in venular blood flow and vaso-occlusions and white cell/endothelium interactions in that SCD mouse model^34^. Interestingly, in that same study, the discontinuation of dexamethasone was associated with rebound increases in adhesion molecules and endothelial activation^34^. While we did not examine the effects of corticosteroid discontinuation here, we found that in the Townes mouse, a different humanized SCD model, a six-week course of prednisolone, a conventional corticosteroid, and of vamorolone, a dissociative corticosteroid, decreased white cell counts and hepatic inflammation, thus suggesting that these drugs indeed decreased ongoing inflammation at varying degrees. However, these seemingly beneficial effects of reduced inflammation were coupled with increased transaminases and alkaline phosphatase in all genotypes (control, heterozygotes, and homozygotes), and worsened hepatic necrosis in homozygotes only. These findings in SCD mice, may suggest the possibility that corticosteroid compounds, especially dissociative steroids, could be associated with significant hepatic toxicity in SCD.

It is noteworthy that, while vamorolone and prednisolone increased hepatic necrosis in homozygotes, but not in heterozygotes and control mice, both prednisolone and vamorolone were associated with elevations in transaminases and alkaline phosphatase in all genotypes. Seen as a milder complication of corticosteroid compounds, hepatic cholestasis and gallstone disease are known to occur in conditions associated with increased production of cortisol such as in Cushing syndrome and with administration of conventional corticosteroids to treat inflammatory conditions in clinical settings^35^. In normal C57BL/6 mice, dexamethasone administration is associated with cholestasis, increased bile acids, and transaminase elevation. Furthermore, these effects are related to activation of the glucocorticoid receptor as glucocorticoid receptor antagonists, and hepatic down regulation of receptors expression ameliorates hepatic cholestasis during corticosteroid administration^35^. Here we found that both prednisolone and vamorolone were associated with increased transaminases and alkaline phosphatase levels in controls, heterozygous and homozygous mice. These data suggest that, akin to conventional corticosteroids, dissociative corticosteroid compounds can also lead to elevation in transaminases and alkaline phosphatase during its administration in control and heterozygotes and homozygotes sickle cell mice. Therefore, these data suggest that liver function tests should be monitored during prolonged use of corticosteroid compounds, whether using conventional or dissociative corticosteroid drugs.

In rats, researchers have shown that chronic prednisolone treatment damages hepatocytes, producing injuries characterized by hepatocellular necrosis and apoptosis^36^. These findings are associated with impairment of NF-ĸB and of inducible nitric oxide synthase responses, elevations in nitric oxide production, and elevations in nitrated protein in liver tissue as suggested by increases in nitrotyrosine levels^36^. In this investigation, we found that prednisolone- and vamorolone-treated homozygous SCD mice sustained significantly greater hepatic toxicity than did controls or heterozygotes. It is noteworthy that in SCD, nitric oxide synthases are uncoupled, nitric oxide availability is decreased, and ongoing inflammation is present^37^. Further, we showed that during basal conditions (vehicle-treatment) homozygous mice have evidence of significant liver injury with signs of hepatic inflammation and hepatic necrosis, phenotypes that can be seen in humans with SCD^38^. Taken together, these findings suggest that in conditions where there is preexisting liver disease as well as altered nitric oxide biology (e.g. homozygous mice), such as in SCD, there is a possibility of increased susceptibility to corticosteroid-induced hepatic toxicity, and this toxicity is greater with the use of dissociative corticosteroid compounds.

Splenomegaly is one of the phenotypic characteristics of the Townes SCD mouse model and results from extra medullary hematopoiesis in response to anemia^27–30^. We found that both prednisolone and vamorolone (to a lesser degree) decreased relative spleen size. This effect was coupled with significant decreases in white blood cell counts and lymphocytes but with no significant changes in red cell counts, hemoglobin, or hematocrit. Notably, decreases in white cell counts, hepatic inflammation, and spleen size were milder in vamorolone-treated animals compared to prednisolone-treated mice. This suggests that at equipotent doses to those of the conventional corticosteroids, the effects of vamorolone, a dissociative corticosteroid, on hematopoiesis and organ inflammation are milder than those of prednisolone in SCD.

The results presented here are contrary to our hypothesis that conventional and dissociative corticosteroid compounds would ameliorate the nociception phenotype in SCD mice. The findings that corticosteroids did not alter the nociception phenotype might suggest that at least in SCD mice, inflammation might not the primary driver of the previously described hyperalgesia in those animals^28,29^. Further, the fact that SCD patients treated with corticosteroids typically suffer adverse events related to vaso-occlusion (increase hospitalization for pain, acute chest syndrome, strokes) might suggest that corticosteroids may alter blood rheology, a hypothesis, which is worthy of testing in future studies. Further, while this preclinical study examined only one level of equipotent doses for prednisolone and vamorolone, which have been shown to be beneficial in mouse models of brain tumor and of inflammatory bowel disease^23,26^, it is possible that different doses could have yielded different results. In fact, vamorolone was shown to be well tolerated in healthy adults at doses of up to 20 mg/kg/day in completed phase 1 clinical trials and is in phase 2 clinical trials in patients with Duchenne muscular dystrophy (NCT02760264). Nevertheless, both conventional (prednisolone) and dissociative corticosteroids (vamorolone) were associated with significant hepatic toxicity manifested by elevations in transaminases, alkaline phosphatase, and increases in liver necrosis. This study describes a previously unknown manifestation of corticosteroid compound-associated toxicity in SCD mice and suggests that liver function tests should be monitored during corticosteroid therapy in SCD.

## Materials and Methods

### Animals

The preclinical trial protocol was approved by the Children’s Research Institute Animal Care and Use Committees and all experiments were performed in accordance with the NIH Guide for the Care and Use of Laboratory Animals^39^. In this investigation, we used Townes sickle cell disease mice^27–32^, which are engineered to express no murine α or β hemoglobin, as murine genes were knocked-out, and instead, to express human hemoglobin as human hemoglobin genes were knocked-in^31,32^. All genotypes carry two alleles of the human α- (hα) globin gene. Control Townes (hα/hα::β^A^/β^A^) mice carry wild-type human β^A^ genes, heterozygotes (hα/hα::β^A^/β^S^) carry one allele of the wild-type human hemoglobin beta (β^A^) gene and one of the sickle hemoglobin beta (β^S^), and homozygotes (hα/hα::β^S^/β^S^) carry two alleles of β^S^. All animals were genotyped as previously described^27^. Homozygous Townes have been shown to recapitulate the altered nociception phenotype, hematologic abnormalities, and organ dysfunction of human SCD^27–30^.

### Preclinical trial

We examined the effects of conventional (prednisolone) and of dissociative (vamorolone) corticosteroid compounds compared with vehicle on the nocifensive behavior phenotype, hematologic profile, and organ dysfunction in heterozygotes, homozygotes, and control Townes SCD mice. A balanced number of age-matched (8–12 weeks) male and female mice from each of the three genotypes were treated daily with vamorolone, prednisolone, or vehicle (cherry syrup) (ReveraGen BioPharma, Rockville, MD) for six weeks. Behavior outcome measurements were obtained before and after completion of six weeks of treatment while animals were receiving the experimental drugs, whereas all other outcome measurements were obtained after treatment only. Investigators who treated animals daily, measured behavior outcomes, and procured organs, were blinded to animals’ genotype and treatment assignments. In this preclinical trial, there were 9 treatment groups including all three genotypes (control, heterozygotes, and homozygotes) and all three experimental drugs (vehicle, prednisolone, or vamorolone). In order to avoid potential confounding effects related to drug preparation or investigator administering drugs, at any given week there were animals from all treatment groups

### Experimental therapies: vamorolone, prednisolone, and vehicle

All treatment drugs were supplied by ReveraGen and were administered daily at doses of 30 mg/kg for vamorolone and prednisolone suspended in cherry syrup (vehicle). All drugs were administered at a volume of 10 µl per g body weight orally while animals were gently held and given syrup drops using a micropipette. Vamorolone and prednisolone doses were selected based on previous preclinical studies of vamorolone showing potent anti-inflammatory effects^23,26^.

### Behavior Studies

In order to minimize variability, behavior assays were conducted by the same investigator between 9 AM and 2 PM and at any given week, included animals from all experimental groups. We examined nocifensive behavior in response to “phasic pain”^40^ using thermal (hotplate and tail flick latencies) and electrical stimuli (sensory fiber interrogation)^40^. Only one of each behavior testing paradigm was conducted per day and the investigator conducting quantitative sensory testing was unaware of the animals’ genotype and treatment.

### Nocifensive response to thermal stimulation

To evaluate nocifensive response to noxious heat, mice were placed on a hotplate (Harvard Apparatus, Holliston, MA) and time to display pain-avoiding behaviors (jumping, stomping or repeated lifting or licking of hind or front paws) was measured^40^. The hotplate temperature was set (55 °C) in accordance with previous studies of SCD^28–30^. Animals were allowed to stay on the hotplate for a maximum of 30 seconds to avoid injury.

### Tail-flick latency

Mice were gently held in a mouse holder and the middle third of the tail was placed over a radiant heat source (Ugo Basile, Varese, Italy) with infrared intensity set at 20%. Latency to withdraw from the heat source (tail flick) was measured to the nearest 0.1 s. Withdrawal of the tail stopped the stimulus, latency to tail flick was recorded automatically, and the cut-off time was set at 15 s. The overall tail flick latency was an average of three measurements obtained at least 1 min apart^40^.

### Nocifensive response and nerve fiber interrogation using sine wave electrical stimulation

In order to evaluate specific somatosensory fibers, we used a sine-wave electrical stimulation paradigm using three frequencies: 5, 250 and 2000 Hz, which preferentially stimulate C, Aδ, and Aβ fibers respectively as previously described^41–43^. Briefly, electrical stimuli generated by a neurostimulator (Neurotron, Inc, Baltimore, MD) were delivered to the tail of gently restrained mice. Stimuli at different frequencies (5, 250 and 2000 Hz) were delivered at increasing intensities, lasted one second and were set on a 50% duty cycle (each 1 s stimulus is followed by a one-second stimulus-free interval). Between stimulations at different frequencies, animals rested for one-minute. The nocifensive behavior outcome was vocalization and its occurrence prompted termination of the stimulus. For each frequency, the electrical stimulus amperage that elicited audible vocalization or the maximum amperage delivered was defined as the current threshold^42–44^. Current thresholds for each frequency were the average of five consecutive measurements obtained in response to 2000, 250, and 5 Hz sequentially. The current threshold unit of measurement is “unit” (U), which corresponds to 100 times the amperage that elicited audible vocalization.

### Hematologic and biochemical parameters

Blood was collected from anesthetized animals via cardiac puncture into heparin-coated syringes. Complete blood cell counts were performed using the Hemavet blood counter (Drew Scientific, Dallas, TX) as previously described^27,30^. After complete blood count, plasma was isolated and frozen until measurement of alkaline phosphatase (ALK), alanine aminotransferase (ALT), and aspartate aminotransferase (AST) in our clinical laboratory. We also measured malondialdehyde formation in liver homogenates, a measure of free radical oxidation of polyunsaturated fatty acids, using the thiobarbituric acid reactive substance (TBARS) fluorometric assay kit (BioAssay Systems, Hayward, CA) following the manufacturer’s protocol^45^.

### Histopathology

Following anesthesia and exsanguination, liver samples were collected and fixed in 10% buffered formalin for histological evaluation. Samples from all surviving animals were embedded in paraffin and 5 µm sections were stained with hematoxylin and eosin (H&E). Slides were digitally scanned using an Aperio XT scanner and reviewed using Aperio ImageScope software. The “pen” tool of the ImageScope software was used to designate regions of interest (ROI), and subsequently used to determine the percentage of liver necrosis. An investigator blinded to genotype and treatment assignments annotated the images by outlining the necrotic areas and the total area of tissue on each slide. ImageScope software generated measurements for the area of outlined tissue in µm^2^ and the total percentage of liver necrosis per animal was calculated.

### Statistical analysis

All outcomes evaluated here were continuous, quantitative traits and were assessed for normality using both a Shapiro-Wilk normality test and visual inspection of histograms.

Several data transformations were used on those outcomes that were not normally distributed. Once transformation was applied, normality was verified. Three different data transformations were used here, log, square root, and cubic as appropriate for the outcome. Specifically, a log transformation was applied to the number of white and red blood cells, mean corpuscular volume and mean corpuscular volume. A square root transformation was applied to the number of neutrophils and lymphocytes and to the red cell distribution width. Lastly, a cubic transformation was applied to hemoglobin and hematocrit values.

Comparisons of all outcomes except hepatic necrosis were performed using ANCOVA models with main effects of treatment group and genotype and a treatment*genotype interaction term. Sex was included as a covariate in all models. For nociception outcomes we used the percent change from baseline as the dependent variable and included an additional covariate of baseline value. Where ANCOVA models showed a statistically significant main effect of treatment and/or genotype, *post-hoc* comparisons between levels of treatment and/or genotype were performed and resulting p-value adjusted for multiple comparisons using the Sidak method.

Comparisons of hepatic necrosis used different statistical method due to all control and heterozygote mice having no evidence of necrosis. Homozygous mice were compared to control mice, within each treatment group, using a Wilcoxon rank-sum test for non-normally distributed data. Homozygous mice were not specifically compared to heterozygous mice as this comparison was unnecessary given both the control and heterozygous mice showing no necrosis. In addition, levels of hepatic necrosis were compared among the three treatment groups in the homozygous mice only, again using a Wilcoxon rank-sum test.

All analyses were performed using Stata V15 (College Station, TX). The significance level of each test was set at 0.05 and no adjustments for multiple testing of multiple outcomes were performed. The datasets generated during and/or analyzed during the current study are available from the corresponding author on reasonable request.
